# Fisetin as a Senotherapeutic Agent: Biopharmaceutical Properties and Crosstalk between Cell Senescence and Neuroprotection

**DOI:** 10.3390/molecules27030738

**Published:** 2022-01-23

**Authors:** Osama Elsallabi, Antonia Patruno, Mirko Pesce, Amelia Cataldi, Simone Carradori, Marialucia Gallorini

**Affiliations:** 1 Department of Medicine and Science of Aging, University “G. d’Annunzio” of Chieti Pescara, 66100 Chieti, Italy; osama.elsallabi@unich.it (O.E.); antonia.patruno@unich.it (A.P.); mirko.pesce@unich.it (M.P.); 2 Department of Biosciences and Nutrition, Karolinska Institutet, SE-141 57 Huddinge, Sweden; 3 Department of Pharmacy, University “G. d’Annunzio” of Chieti-Pescara, 66100 Chieti, Italy; amelia.cataldi@unich.it (A.C.); marialucia.gallorini@unich.it (M.G.)

**Keywords:** fisetin, senescence, neuroinflammation, neuroprotection, senolytic drug, in silico evaluation

## Abstract

Like other organs, brain functions diminish with age. Furthermore, for a variety of neurological disorders—including Alzheimer’s disease—age is one of the higher-risk factors. Since in many Western countries the average age is increasing, determining approaches for decreasing the effects of aging on brain function is taking on a new urgency. Neuroinflammation and oxidative stress are two convoluted key factors in brain aging and chronic neurodegenerative diseases. The diverseness of factors, causing an age-related decrease in brain functions, requires identifying small molecules that have multiple biological activities that can affect all these factors. One great source of these small molecules is related to polyphenolic flavonoids. Recently, 3,3′,4′,7-tetrahydroxyflavone (fisetin) has been reported as a potent senotherapeutic capable of extending lifespan by reducing peroxidation levels and enhancing antioxidant cell responses. The neuroprotective effects of fisetin have been shown in several in vitro and in vivo models of neurological disorders due to its actions on multiple pathways associated with different neurological disorders. The present work aims to collect the most recent achievements related to the antioxidant and neuroprotective effects of fisetin. Moreover, in silico pharmacokinetics, pharmacodynamics, and toxicity of fisetin are also comprehensively described along with emerging novel drug delivery strategies for the amelioration of this flavonol bioavailability and chemical stability.

## 1. Introduction

Aging is a highly malleable process that can be modulated in different ways, such as caloric restriction, intermittent fasting, exercise, and a plant-based diet rich in phytochemicals [1]. The use of bioactive compounds to eliminate senescent cells has recently emerged as a promising approach to delay aging and reduce the severity of chronic diseases. Among others, neurodegenerative age-related diseases—including Alzheimer’s disease (AD) and Parkinson’s disease (PD)—currently affect people worldwide. Unfortunately, no treatments are currently available to prevent disease development and progression. Plant-derived flavonoids have a wide range of activities that could make them particularly effective for blocking the age-associated toxicity pathways associated with neurodegenerative diseases [2]. Fisetin has recently emerged as a potential anti-inflammatory, chemopreventive, chemotherapeutic, and senotherapeutic agent, thus making it a good candidate for the treatment of neurodegenerative diseases [3].

Fisetin is a 3,7,3′,4′-tetrahydroxyflavone widely found as glycosides or as aglycone in various edible fruits and vegetables such as strawberries (*Fragaria* sp. 160 μg/g), apples (*Malus* sp. 26.9 μg/g), persimmons (*Diospyros* sp. 10.6 μg/g), Lotus roots (*Nelumbo* sp. 5.8 μg/g), onions (*Allium* sp. 4.8 μg/g), grapes (*Vitis* sp. 3.9 μg/g), kiwi fruits (*Actinidia* sp. 2.0 μg/g), peaches (*Prunus* sp. 0.6 μg/g), cucumbers (*Cucumis* sp. 0.1 μg/g), and tomatoes (*Solanum* sp. 0.1 μg/g). The average human daily intake is estimated to be around 0.4 mg [4]. It has been recently demonstrated that this natural compound could modulate different pleiotropic pathways (phosphatidylinositol-3-kinase/protein kinase B/mammalian target of rapamycin (PI3K/Akt/mTOR) and p38, mitogen-activated protein kinases (MAPK)-dependent nuclear factor kappalight-chain-enhancer of activated B cells (NF-κB)) exerting a large plethora of biological effects including anti-inflammatory, hypolipidemic, hypoglycemic, antioxidant, neuroprotective, antiangiogenic, and antitumor ones [5,6,7].

The present review aims at collecting the most recent achievements related to the antioxidant and neuroprotective effects of fisetin. Moreover, pharmacokinetics and pharmacodynamics of fisetin and fisetin-related metabolites are also comprehensively described.

## 2. Crossroads of Neuroinflammation and Neurodegenerative Disorders

### 2.1. Neurodegenerative Disorders

Neurodegenerative disorders are classified by their clinical features, with few patients showing pure syndromes and most having mixed clinical presentations. Most neurodegenerative diseases show common physiological signs characterized by progressive neuronal dysfunction and death triggered by several dysfunctional physiological processes, such as protein abnormalities, proteotoxic stress, and its attendant abnormalities in ubiquitin–proteasomal and autophagosomal/lysosomal systems, oxidative stress, programmed cell death, and neuroinflammation [8]. Among protein abnormalities, amyloidosis represents the most common dysfunction in almost all common neurodegenerative diseases. Amyloids are insoluble fibrous proteins and amyloid-like filamentous aggregates are mostly within the cytoplasm of neurons and glia under neurodegenerative conditions. The most common aggregate is referred to β-amyloid or Aβ which is the main pathological dysfunction leading to Alzheimer’s disease. AD is nowadays the commonest neurodegenerative disease and the most frequent cause of dementia, affecting 50 million people worldwide in 2018 [9]. Currently, there are only two classes of approved drugs to treat AD, including cholinesterase inhibitors and antagonists to N-methyl D-aspartate (NMDA), which are effective only in treating the symptoms but do not cure or prevent the disease.

Neurological disorders are now the leading source of disability in the world, and Parkinson’s disease is the fastest growing of these disorders. Clinically characterized by the cardinal features of rest tremor, bradykinesia, rigidity and postural instability, and a variety of other motor and non-motor symptoms, PD etiopathogenesis includes the accumulation of intracytoplasmic bodies named “Lewy’s bodies” and dopamine deficiency. The underlying key molecular mechanisms include α-synuclein misfolding and aggregation, mitochondrial dysfunction, impairment of protein clearance, neuroinflammation, and oxidative stress [10]. Individuals with mild motor-predominant PD (49–53%) have mild symptoms, a good response to dopaminergic medications (e.g., carbidopa–levodopa, dopamine agonists, monoamine oxidase B inhibitors [11]), and slower disease progression. Dopamine-based therapies initially help to decrease motor symptoms. Contrariwise, non-motor symptoms require non-dopaminergic approaches (e.g., selective serotonin reuptake inhibitors for psychiatric symptoms, cholinesterase inhibitors for cognition). No disease-modifying pharmacologic treatments are currently available [12].

In this light, alternative and innovative therapeutic approaches for neurodegenerative diseases are urgently needed. Since neuroinflammation is a common feature in most pathologies leading to neurodegeneration, a valid approach to adjuvate traditional treatments must counteract the molecular mechanisms underlying inflammation in the CNS.

### 2.2. The Interplay between Neuroinflammation and Neurodegeneration

**Figure 1:**
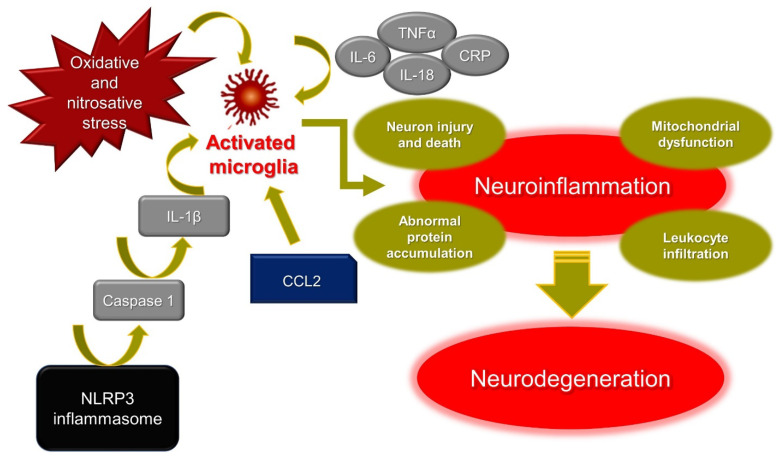
Mechanisms of neuroinflammation. IL-6: interleukin-6; IL-18: interleukin-18; IL-1β: interleukin-1β; TNFα: tumor necrosis factor α; CRP: C-reactive protein; CCL2: C-C motif chemokine ligand 2; NLRP3: NOD-like receptor protein 3.

The term neuroinflammation defines an inflammatory cell or tissue response within the brain or spinal cord. Resident central nervous system (CNS) glia (microglia and astrocytes), endothelial cells, and immune cells produce cytokines, chemokines, reactive oxygen species (ROS), and secondary messengers to mediate neuroinflammation processes (Figure 1). As for general inflammation, the degree of neuroinflammation is strongly influenced by the cell context, duration, and course of the primary stimulus or insult [13]. Microglia contains the main cell types implicated in neuroinflammation. Indeed, most of the CNS innate immune capacity resides there and thus microglia has an active role in immune surveillance [14].

Under infection or disease, microglia become “activated” and resident cells function as inflammatory mediators. Activated cells in microglia rapidly produce pro-inflammatory cytokines and chemokines, facilitating the recruitment of leukocytes in the brain [15]. Neuroinflammation is mediated by several key cytokines, interleukin (IL)-1β, IL-18, IL-6, tumor necrosis factor (TNF)α, chemokines (CCL2, CCL5, CXCL1), secondary messengers (NO and prostaglandins), and ROS. In general, CNS benefits from fine and tight modulation of microglial activation which is intended to protect the host organism. Nonetheless, chronic microglial activation due to amplified or exaggerated cytokine production can lead to robust pathological changes and neurodegeneration [14].

IL-1β and IL-18 have prominent functions in the CNS, and therefore many nervous cell types express their cognate receptors which mediate inflammatory signaling cascades that may contribute to neuronal injury and cell death. Increased levels of IL-1β and IL-18 are often observed upon CNS infection, brain injury, and neurodegenerative diseases [16]. It has been reported that pro-inflammatory markers, including IL-1β, IL-6, TNF-α, and C-Reactive Protein (CRP), are significantly higher in the elderly with depression and AD [17]. NOD-like receptor protein 3 (NLRP3) inflammasomes play a crucial role in PD via caspase 1 activation, inducing IL-1β maturation and leading to neuronal pyroptosis. Furthermore, the abnormal aggregation of α-synuclein, which is the main pathogenesis factor of PD, also activates NLRP3 inflammasomes [18].

Recently, chemokines have become a focus in the context of the development and treatment of brain diseases. Among them, chemokine CCL2 and its main receptor CCR2 have become candidate mediators of abnormal brain–immune-system dialogue in depression [19]. It has also been reported that an atypical chemokine receptor CCL2 binding, supports sustained inflammation and increased AD risk [20].

Mitochondrial dysfunction and oxidative and nitroxidative stress have emerged as major contributors to degeneration of dopaminergic neurons in PD [21], AD [22] and in general in neurodegenerative diseases. One of the histopathological hallmarks of AD is the formation of extracellular senile plaques of the amyloid-β (Aβ) peptide aggregated with metal ions such as copper, iron, or zinc. Redox-active metal ions, such as copper, for example, can catalyze the production of ROS when bound to the Aβ. It has been reported that the ROS produced thus may contribute to oxidative damage on both the Aβ peptide itself and on the surrounding molecule [23].

To date, available therapy options for neurodegenerative diseases have not shown fully satisfactory results in finding a curative or disease-modifying treatment and many drug candidates have failed in clinical development. Thus, novel approaches in the prevention of neurodegenerative processes could be necessary. One possible solution could be a combination of easily and cheaply available food-derived substances (so-called “nutraceuticals”) with a low profile of adverse reactions, aiming at supporting canonical therapies, targeting the multiple involved pro-inflammatory mediators implicated [24]. Natural compounds, such as flavonoids, own neuroprotective potential related to their ability to modulate the inflammatory responses involved in neurodegenerative diseases in the microglia [25]. Fisetin is one of the most common and bioactive flavonoids which possesses potential neuroprotective effects. It has been reported that fisetin enhances learning and memory, decreases neuronal aging-related cell death, and suppresses oxidative stress [26]. Most important for our discussion, it has been reported that, on ten natural flavonoids tested in vivo, fisetin was the most potent senolytic compound, reducing senescence markers in progeroid and old mice [27]. In this work, it has been therefore demonstrated that fisetin reduced senescence in a subset of cells in murine and human adipose tissue, demonstrating cell-type specificity. Moreover, the fisetin administration to wild-type mice late in life restored tissue homeostasis, reduced age-related pathology, and extended median and maximum lifespan, disclosing that fisetin is a promising senotherapeutic compound both in mice and humans. Since aging is deemed one of the major risk factors which leads to various chronic diseases and disabilities such as neurodegenerative diseases [28,29], in the next paragraphs the crossroads between senescence and neurodegeneration are highlighted, as well as the molecular mechanisms underlying the effects of fisetin as a senolytic drug for neuroprotection.

## 3. Fisetin as a Senolytic Drug: Crosstalk between Neuroprotection and Senescence

One of the characteristics of normal aging is a decrease in both cognitive and motor functions, resulting in changes in learning and memory as well as deficits in balance and coordination. Furthermore, for a variety of neurological disorders (including AD), age is one of the higher risk factors. Since in many Western countries the average age is increasing, determining approaches to decrease the effects of aging on brain function is taking on a new urgency. However, to choose among possible approaches, it is first necessary to identify the factors which contribute to the decrease in brain function with age. Recent evidence suggests an association between cellular senescence and age-related diseases. Senescent cells are characterized by an irreversible replicative arrest, resistance to apoptosis, increased ROS generation, metabolic shifts, and acquisition of a pro-inflammatory senescence-associated secretory phenotype (SASP). The latter can accumulate in various tissue sites such as the CNS, suggesting that it can contribute significantly to the cognitive decline characteristic of neurological disease.

In order, focusing attention and therapeutic interventions on the biological mechanisms underlying aging and particularly on the reduction of senescent cells using senolytic drugs could be important to alleviate age-related diseases such as neurodegenerative diseases.

### 3.1. Senescence Mechanisms

Cellular senescence occurrence is widely reported as the first sign of aging [30]. In 1961 the first experimental evidence was highlighted for cellular aging in vitro. Leonard Hayflick and Paul Moorhead established in a ground-breaking study that normal diploid human fibroblasts have a restricted ability to replicate before they enter a stage known as replicative senescence [31]. Subsequently, this specific form of senescence (known as replicative senescence) was connected to telomere attrition, which promotes tumorigenesis and chromosomal instability, consistent with the original hypothesis that uncontrolled cell growth of damaged cells is counteracted via senescence [32]. Permanent cell cycle arrest in senescent cells plays a fundamental role in anticancer machinery [33]. Several stressors can induce senescence; for instance, ROS, DNA damage, strong mitogenic/oncogenic signaling, depletion of specific tumor suppressors, arrested DNA replication, and chromatin disruption [34,35,36,37,38,39,40].

However, the senescent cell cycle arrest is governed by tumor-suppressive pathways, and it is organized through p53/p21 and p16INK4A/retinoblastoma (RB) [41]. The DNA damage response (DDR) implies the occurrence of DNA double-strand breaks and uncapped telomeres, which results in post-translational phosphorylation events via ATM and ATR serine/threonine protein kinases. These events lead to p53-dependent DNA stabilization or to p14ARF-mediated inhibition of the ubiquitin ligase MDM2 upon hindering degradation of p53 [42,43]. The transcription of the cyclin-dependent kinase inhibitor (CDKi) p21 takes place once p53 is stabilized, triggering transient cell cycle arrest [44]. Next, a permanent arrest is maintained by the transcriptional upregulation of p16INK4A mediated by p38 [45] and/or ERK signaling [46]. Once established, a permanent blockage of the S phase entry occurs, due to the inhibition of the activity of both CDK4 and CDK6 via p16INK4A, thus leading to RB hyperphosphorylation. Substantially, the expression of p16INK4A increases with aging in different types of tissues and it is considered as a biomarker of natural aging [47].

The number of senescent cells increases with age in vivo, and one of the most important methods to detect them is measuring the senescence-associated β-galactosidase activity (SA β-gal) at pH 6.0 [48]. Senescent cells are considered to apply their adverse effects with aging and in age-related diseases to a certain extent via the SASP, which consists of multiple growth factors, proteases, and inflammatory cytokines [49].

Cell fate depends on various incentives, such as impairment of specific tumor suppressors or oncogene activation. As a strategy for preventing neoplastic transformation, cells might undergo senescence or apoptosis. p53 is a major key regulator of this cell decision based on the level of its activation. Excessive p53 signaling leads to the overexpression of proapoptotic modulators including NOXA and PUMA, which causes cell death. Milder “senescence-inducing” stressors stimulate p53-mediated transcription of the CDKi p21 and BCL-2 anti-apoptotic family members, including BCL-W, BCL-2, and BCL-XL [50]. These proteins bind to proapoptotic proteins with BH3 domains, preventing mitochondrial outer membrane permeabilization and apoptosis [51]. While transient low-grade p53 activation aids repair, persistent activation can lead to cell senescence. These situations cause antiapoptotic proteins to be produced to promote survival, which might explain why senescent cells grow in vitro with passaging and in vivo with increasing age [52].

### 3.2. CNS Senescence

Aging drives chronic brain inflammation in a gradual manner and influences the homeostasis of all brain cell types, including neurons, microglia, astrocytes, oligodendrocytes, and endothelial cells [53]. The accumulation of inflammatory cytokines occurs without clear evidence of the pathogen. This leads to decreased pre- and postsynaptic densities alongside a total decrease in synapses and dendritic spines, which induce memory loss and cognitive deficits in old individuals. However, a typical feature of many neurodegenerative disorders is chronic inflammation levels associated with aging, which indicates that the normal aging process and pathological modification have mutual proinflammatory pathways [52].

#### 3.2.1. Neurons

In vivo, terminally differentiated neurons may show signs of senescence correlated with advanced age. Additionally, it has been demonstrated that in neurodegenerative diseases, for instance in AD patients, nervous cells have shortened telomeres [54]. On the contrary, PD patients do not exhibit these alterations [55]. Furthermore, in 2012, Jurk et al. illustrated more extensive and comprehensive evidence for neuronal senescence [56]. For the first time, this study demonstrated that Purkinje and cortical neurons in 32-month-old mice have various cell senescent hallmarks, including the most common senescent-driven pathways in a p21/CDKN1A dependent manner [56]. These findings demonstrated that neuronal cells have similar senescent features compared to proliferating cells. In 2015, a study on human brains verified neuronal senescence in mice and rats, demonstrating p16/CDKN2A expression in pyramidal neurons in the brains of old individuals (above 77 years) [57]. Furthermore, senescent neurons secrete pro-inflammatory cytokines which may play a key role at the beginning of neurodegeneration. It has been demonstrated that neurons in AD patients have senescent features such as augmented p38/MAPK activity, TGF mRNA expression, IL-6 expression, and p16/CDKN2A expression and that higher SASP activity can trigger senescence in surrounding cells in a paracrine way [45].

#### 3.2.2. Microglia

Microglia, which originated during development in the bone marrow, are the fundamental immune cells of the brain and are functionally macrophage-like [58]. Normally quiescent microglia become activated as a reaction to invading microorganisms or injury, release different growth factors and cytokines, and induce phagocytosis [59]. Senescence-upregulated proinflammatory cytokines, including IL-6, IL-1β, and TNF-α, were highly expressed in microglia from aged mice [49,60,61]. Furthermore, shorter telomeres are present in naturally aged microglia [62]. Chronically activated microglia demonstrate different features of senescence, including growth arrest, heterochromatic foci formation, and SA β-gal activity, among others [63]. Interestingly, TGF-β levels in plasma and cerebrospinal fluid were observed to be increased in Alzheimer’s patients. However, unpaired TGF-β1-SMAD3 signaling was expressed in senescent microglia, which involved aberrant activation and decrease of phagocytic Aβ peptide activity [64].

#### 3.2.3. Astrocytes

The brain is one of the richest body organs with astrocytes, which were formerly thought to be non-functional neural network fillers. Nevertheless, as time passes and technology improves, the significance of these cells in a variety of biological processes has been established. Astrocytes are crucial for maintaining homeostasis by balancing osmotic balance [65]. Furthermore, astrocytes have been shown to have a role in synaptic transmission by being adjacent to neurons, which is a component of the tripartite synapse and enhances neurotransmission between pre- and postsynaptic parts. Moreover, because of their participation in innate immunity, astrocytes play a role in the defense against brain inflammation such as neurodegeneration, trauma, and infection [66].

### 3.3. Senolytic Drugs

To our knowledge, during pathological conditions senescent cells may accumulate with age in the brain. Currently, there are no methods or strategies for identifying, isolating, and eliminating senescent cells, and distinguishing their mechanistic involvement in neuropathology is exceedingly challenging. Until now, identifying senescent cells has relied on a mixed group of markers that are not specific, to verify that the alterations are not caused by other factors. It was observed that the use of genetically modified mice, through genetic inactivation of p16INK4A or the elimination of senescent cells, may impede cells from becoming senescent, which causes neuropathology. However, few relevant researchers have applied these techniques to neurodegenerative pathologies, and p16INK4A mice with genetic inactivation are extremely tumor prone and die before cognitive impairment is noticed. To deal with this problem, two genetically engineered mice models were established. The INK-ATTAC transgenic mouse uses a 2.6-kb p16INK4A promoter region to induce drug-responsive caspase-8 expression when the drug AP20187 is introduced to mice [67]. Another useful model, the p16-3MR transgenic model, expresses a trimodal reporter construct under the control of a bacterial artificial chromosome that contains the whole p16INK4A promoter. Monomeric red fluorescent protein (mRFP), synthetic Renilla luciferase, and a shortened herpes simplex virus thymidine kinase make up this reporter (HSV-TK). When ganciclovir is given to p16INK4A-expressing cells, HSV-TK turns it into a hazardous DNA chain terminator, causing senescent cells to die through mitochondrial-dependent cell death [68,69]. These models helped to improve our understanding of senescent cell role in various age-related diseases. Senescent cells (SCs) might be removed for therapeutic benefit without causing detrimental side effects. This is a major finding that has paved the way for the creation of drugs and strategies that target SNC, particularly for the prevention and finding a cure for age-related diseases [67,70,71]. Several approaches have been developed aiming at the clearance of senescent cells and the reduction of SASP, e.g., the development of genetic clearance models (transgenic animals) and the identification and development of drugs referred to as “senotherapeutics”. Currently, the identification of senotherapeutic drugs represents a promising area of research for new therapies. Even if the knowledge of their potential molecular targets and their mechanisms of action are still limited, senotherapeutics are classified as (i) senolytics agents selectively targeting senescent cells to eliminate them; (ii) senomorphics agents, which create a controlled environment by suppressing markers of senescence and targeting a specific component of SASP. As a consequence, the functions and morphology of senescence cell are modulated by reverting it or by slowing the progression of cells towards the senescent phenotype; (iii) immune-system mediators of the clearance of SCs. Tests conducted to investigate the mechanism of action of different senotherapeutic agents have revealed that they can be classified as dual agents (senolytic and senomorphic), in a cell-specific and concentration-dependent manner, obviously in correlation to the heterogenicity of senescent cells [72].

Senolysis, rather than SASP suppression, has the most therapeutic potential for two main reasons. First, the permanent removal of SCs results in the long-term elimination of harmful SASP components. Second, there is no probability of tumorigenic “escape” from senescence once a SC has been removed [73].

Senolytics were the first senotherapeutic agents to be effectively evaluated in preclinical in vivo models, and there are already multiple senolytic drugs available. Many of these drug targets increased anti-apoptosis mechanisms in SCs, including signaling via the BCL2 protein family (BCL2, BCLXL, and BCLW). These proteins bind to pro-apoptotic BCL2 family members and functionally deactivate them [51,74]. BCL2 proteins that are pro-apoptotic, activate the BAX and BCL2 homologous antagonist/killer (BAK) proteins, inducing cytochrome c release and programmed cell death [75].

### 3.4. Fisetin as Senolytic Drug in the CNS

Fisetin is a senolytic flavonoid that is found in nature. It functions by suppressing BCL-2 family members such as BCL-xL, as well as HIF-α and other components of the SCAP network [27]. Moreover, Singh and colleagues evaluated the neuroprotection properties of fisetin in induced, accelerated, and natural aging models of rat. Interestingly, it was found that fisetin significantly reduced the peroxidation levels and increased antioxidants responses. Furthermore, mitochondrial membrane depolarization and apoptotic cell death were attenuated in aging rat brain. At the genetic level, fisetin increased the expression of autophagy genes (Atg-3 and Beclin-1) and NSE and Ngb sirtuin-1 neuronal markers and decreased the expression of IL-1β, TNF-α, and Sirt-2 inflammatory genes in the aging brain [76]. Interestingly, Cho et and coworkers [77] found that fisetin significantly decreased neuronal defects at days 5 and 10 in a *Caenorhabditis elegans* model. Indeed, worms exposed to fisetin exhibited decreased anterior lateral microtubule (ALM) and posterior lateral microtubule (PLM) morphological defects at day 5 compared to the DMSO control.

It has been furthermore reported that treatment with fisetin attenuated brain edema and cell apoptosis in intracerebral hemorrhage (ICH) mice, which exhibited a significant increase in the modified neurologic severity scores (mNSS). Fisetin attenuated severe brain deficit by decreasing levels of proinflammatory cytokines. Moreover, diminution of NF-κB signaling was reported after fisetin treatment [78]. However, a recent study demonstrated an increase in p16Ink4a senescent cells in an age-dependent manner, which was more noticeable in microglia and oligodendrocyte progenitor cells. In aged mice, with p16Ink4a-positive senescent cells, they were genetically eliminated upon treatment with two specific drugs or with the senolytic cocktail (dasatinib and quercetin). It was demonstrated that both strategies resulted in a decrease in p16Ink4a particularly in the microglial population, resulting in reduced microglial activation and reduced expression of SASP factors. Substantially, these two methods significantly improved cognitive function in aged mice, suggesting that senolytic interventions might be a potential therapeutic approach to mitigate age-associated cognitive impairment [79].

One of the physiological brain age indicators is the electroencephalograph (EEG). Various sensory–cognitive inputs modify brain oscillatory waves from neural tissue. Furthermore, fast-wave oscillations (e.g., 8–12 Hz or 12–28 Hz) are linked to coordination and deal with difficult behavioral tasks. In a recent study, Das and colleagues [80] tested fisetin effects in vivo through cortical spectral power oscillations and multi-unit activity (MUA), finding that in aged rats the relative spectral power of α and β decreased along with the MUA count compared to young ones.

Intriguingly, in vivo study on aging senescence-accelerated prone 8 (SAMP8) mice demonstrated that fisetin prevents cognitive and locomotor deficits. Additionally, three proteins linked to synaptic function were reduced in aged mice compared to young mice, and fisetin treatment imped their reduction almost completely [81]. Moreover, in another study on brain mice injected with D-galactose (D-gal) to accelerate senescence, fisetin significantly reduced ROS generation induced by D-galactose, along with neuroinflammation-related pathways and pro-apoptotic markers [82].

## 4. Fisetin: In Silico Evaluation of Pharmacodynamics, Pharmacokinetics, and Toxicity

Fisetin, chemically known as 2-(3,4-dihydroxy)-3,7-dihydroxy-4*H*-1-benzopyran-4-one, has a peculiar flavone scaffold like that of the more famous quercetin (see paragraph 3.4). Like other flavonoids, it displays poor aqueous solubility (9.55–10.45 µg/mL at 37 °C) [83] and high affinity to polar organic solvents (2.89 mg/mL in ethanol:water 50/50 at 27 °C), pKa_1_ = 7.27 ± 0.09 and pKa_2_ = 9.44 ± 0.07 [84], and experimental Log*P* of 2.20 [85]; contrariwise, its antioxidant activity is not so efficient despite the presence of a reactive catechol portion [3]. This feature led to the discovery of low stability or bioavailability of this secondary metabolite when tested in cell culture conditions or after in vivo administration, respectively [86]. These unfavorable characteristics can be partially overcome by lipid-based delivery systems such as liposomes, nanoemulsions, nanocochelates, and spherulites [87].

Keeping under consideration that the appraisal of a natural compound as a promising drug is a long and uncertain process, we aimed to evaluate this small molecule by means of fourteen commercially available online machine-learning platforms in terms of pharmacodynamics, pharmacokinetics, and toxicity (Table 1, Table 2 and Table 3). Some data were initially predicted through a tool and then further validated by others if available. We aim to provide new information to better ascertain the biological potential and drug likeness of this widely spread flavonoid, which has been involved till Phase 2 of clinical trials according to the ZINC database (code ZINC39111) (http://zinc15.docking.org, accessed on 30 December 2021) and ChEMBL (https://www.ebi.ac.uk/chembl/compound_report_card/CHEMBL31574/, accessed on 30 December 2021) for the treatment of osteoarthritis of the knee, childhood cancer, and severe acute respiratory syndrome in COVID-19.

Starting with the web-service tool SwissTargetPrediction implemented with SwissSimilarity, we collected data and robust predictive models in Table 1 regarding the highly putative human targets for fisetin with a probability score ≥0.6 [88] (Table 1). Then, we checked if ZINC and ChEMBL could corroborate these data. Moreover, fisetin had no structural similarity to other FDA-approved drugs.

Epigenetic Target Profiler [89] and ChEMBL also proposed the strong affinity for Cyclin-dependent kinase 2 and to a lesser extent to other epigenetic targets (Cyclin-dependent kinase 1, Bromodomain-containing protein 2, Histone acetyltransferase p300, Histone deacetylase 2, Protein kinase 1, Serine-protein kinase ATM).

Lastly, PASS (Prediction of Activity Spectra for Substances, http://www.pharmaexpert.ru/PASSonline/index.php, accessed on 30 December 2021) was developed to predict biological activities with 90% accuracy. Each outcome is characterized by Pa (probability to be active, high if ≥ 0.7) or Pi (probability to be inactive, good if Pa is much higher than Pi). Fisetin displayed discrete cytotoxicity expressed as Pa value of 0.523 and Pi = 0.049 against human Hs 683 cell line (oligodendroglioma).

Drug likeness of fisetin was predicted by determining whether it had properties consistent with being an orally active drug and important in the drug design and development to prevent drug candidates from failing. Calculations were made using different web-service tools such as SwissADME and BOILED-Egg [90,91], pkCSM (http://biosig.unimelb.edu.au/pkcsm/prediction, accessed on 30 December 2021) [92], FAF-Drugs4 (https://fafdrugs4.rpbs.univ-paris-diderot.fr/ [93], accessed on 30 December 2021), OSIRIS Property Explorer (http://www.cheminfo.org/Chemistry/Cheminformatics/Property_explorer/index.html, accessed on 30 December 2021), and PreADMET (v. 2.0, Yonsei University, Seoul, Korea, https://preadmet.bmdrc.kr/adme/, accessed on 30 December 2021) (Table 2). The molecule respected Lipinski’s, Ghose’s, Veber’s, Egans’, and Muegge’s filters with no violation of the drug likeness according to SwissADME and PreADMET. Moreover, it displayed an Abbott bioavailability score of 0.55 and synthetic accessibility score of 3.16 (but it can be also obtained by natural sources).

The recommended range for Caco2 permeability is: <25 poor, >500 great or if *P*_app_ is higher than 8 × 10^−6^ cm/s; for HIA is: >80% high, <25% poor; for BBB: optimum range between −3.0 and −1.2, whereas logBBB >0.3 highly permeable and <−1 poorly permeable; for skin permeability: optimum range between −8.0 and −1.0; for log PS > −2 possible penetration into the CNS, for log PS < −3 not able to penetrate the CNS.

The main metabolites (and their percentages) of fisetin predicted by GLORYx software are 7- (98%), 4′- (97%), and 3′-glucuronides (90%) along with 3′-OCH_3_ (90%) and 3′-sulfated (87%) compounds [94].

Finally, to conclude an exhaustive analysis on fisetin, preliminary data on toxicity influencing the fate of drug candidates were also calculated by ProTox-II [95], CarcinoPred-EL [96], OSIRIS Property Explorer, PreADMET, pkCSM and presented in Table 3. These in silico toxicity models can substitute animal testing, avoiding timewasting and unnecessary cost.

Data collected in silico described fisetin as a good lead compound to be further investigated for specific biological activities, despite the limited aqueous solubility and moderate BBB permeation. This information further justified the loading of fisetin into more complex structures capable of enhancing the delivery and targeting of this bioactive flavonol [96,97,98,99].

## 5. Conclusions

Pathophysiological determinants leading to aging and age-related neurodegenerative diseases are still unknown. The lack of understanding of the mechanisms underlying the onset of neuronal aging and related pathologies represents an obstacle to the development of targeted therapeutic strategies and delays the initiation of clinical translation for a wide range of applications. Recent evidence suggests accumulation of senescent cells with aging may actively contribute to chronic and age-related diseases and conditions such as neurodegenerative diseases. Their detrimental effects appear to be determined by metabolic shifts, increased generation of reactive oxygen species (ROS), and SASP factors. However, the overall significance and mechanistic contribution of these cells to neurodegenerative diseases have not yet been clarified due to the lack of identification and isolation tools. Scientific evidence, showing the efficacy through the elimination of senescent cells in the positive modulation of inflammatory diseases, has aroused an interest in the development of therapeutic strategies for the elimination of senescent cells, indicated as “senotherapy” in the absence of gene modifications. Recently, fisetin was shown to act as a senotherapeutic agent capable of extending lifespan, reducing ROS levels, and enhancing antioxidant cell responses. This neuroprotection has been detected in in vitro and in vivo models associated with different neurological disorders. The present review collected the most recent findings of the antioxidant and neuroprotective effects of fisetin. Furthermore, in silico pharmacokinetics, pharmacodynamics, and toxicity of fisetin were assessed by means of several commercially available web tools. Although senolytics provide a promising avenue for exploration as a treatment for age-related and neurodegenerative diseases, further clinical studies are needed to determine the safety and efficacy of these drugs before routine clinical use can be considered, and then possibly inclusion as agents that are available over the counter as dietary supplements. Determining high-risk populations, reliably identifying early signs of these diseases, recruiting patients at higher risk of developing the disease, and identifying the right time to start treatment are key parameters to consider for the effectiveness of the treatment. Diversifying and exploring new therapeutic ideas is essential, especially for these inherently complex and not very well-understood pathologies.

## Figures and Tables

**Table 1:** In silico prediction of putative pharmacodynamics for fisetin (threshold: probability score ≥ 0.6).

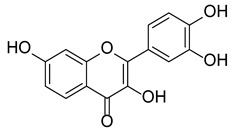		
Human Target	Probability Score (SwissTarget Prediction)	Target Also Confirmed by
Xanthine dehydrogenase	1.0	ZINC
Cyclin-dependent kinases 1, 2, 5 and 6	1.0	ZINC and Epigenetic Target Profiler
Acetylcholinesterase	1.0	ChEMBL
Arachidonate 12 and 15-lipoxygenases	1.0	ZINC
Arginase-1	1.0	
Multidrug resistance-associated protein 1	0.84	
NADPH oxidase 4	0.73	
Aldose reductase	0.73	
Tyrosine-protein kinase receptor	0.73	
Carbonic anhydrases II, VI, VII and XII	0.73	ChEMBL
Arachidonate 5-lipoxygenase	0.73	
Estradiol 17β-dehydrogenase 2	0.73	ChEMBL
P-glycoprotein 1	0.73	
Cytochrome P450 1B1	0.73	
ATP-binding cassette sub-family G member 2	0.73	
Monoamine oxidase A	0.73	ChEMBL
Adenosine A1 receptor	0.73	
Glyoxalase I	0.73	
Tyrosine-protein kinase SYK	0.73	
Glycogen synthase kinase-3β	0.73	
Matrix metalloproteinases 2 and 9	0.73	

**Table 2:** Molecular properties and ADME (absorption, distribution, metabolism, excretion) profile of fisetin.

Parameter		Fisetin	Online Tool
Molecular properties	Molecular weight	286.24	SwissADME, PROTOX-II, and OSIRIS Property Explorer
	Consensus Log*P*	1.55	
	N° of H-bond acceptors	6	
	N° of H-bond donors	4	
	N° of heavy atoms	21	
	N° of aromatic heavy atoms	16	
	Fraction Csp^3^	0.00	
	Molar Refractivity	76.01	
	Topological Polar Surface Area	107.22–111.13 Å^2^	
	Lead likeness	Yes	
	Pan Assay Interference Structures or Structural Alert	yes (catechol)	
	N° rotatable bonds	1	
Absorption	Water solubility (log mol/L)	−3.153	pkCSM
	Caco2 permeability expressed as log *P*_app_ in 10^−6^ cm/s	0.716	
	Intestinal absorption (human) (% adsorbed)	85.46 79.43 (HIA)	pkCSM PreADMET
	Skin permeability (log K_p_)	−6.65 cm/s −2.74 cm/s −4.33 cm/s	SwissADME pkCSM PreADMET
	P-glycoprotein substrate	no yes	SwissADME and PreADMET pkCSM
	P-glycoprotein I inhibitor	no	pkCSM
	P-glycoprotein II inhibitor	no	
	Gastrointestinal absorption	high	BOILED-Egg
Distribution	Volume of distribution at steady state (VDss) (human) (log L/Kg)	0.127	pkCSM
	Fraction unbound (human)	0.045	
	CNS permeability (log PS)	−2.417	
	Blood–brain barrier (BBB) permeability (log BB)	−1.114	
	BBB permeation	no	BOILED-Egg
	In vivo BBB penetration ([brain]/[blood])	0.32 (moderate absorption if in the range 0.1–2.0)	PreADMET
	Caco2 permeability	9.57	
	Pure water solubility (mg/L)	63.3725	
	Plasma protein binding (PPB)	88.73% (weakly bound if <90%)	
Metabolism	CYP2D6 substrate	no	pkCSM and PreADMET
	CYP3A4 substrate	no	pkCSM and PreADMET
	CYP1A2 inhibitor	yes	SwissADME and pkCSM
	CYP2C19 inhibitor	no yes	SwissADME and pkCSM PreADMET
	CYP2C9 inhibitor	no yes	SwissADME and pkCSM PreADMET
	CYP2D6 inhibitor	yes no	SwissADME pkCSM and PreADMET
	CYP3A4 inhibitor	yes	SwissADME, PreADMET and pkCSM
Excretion	Total clearance (log mL/min/Kg)	0.557	pkCSM
	Renal organic cationic transporter (OCT2) substrate	no	pkCSM

*P*_app_: apparent permeability; HIA: human intestinal absorption.

**Table 3:** Prediction of toxicity for fisetin.

Parameter/Target	Fisetin (Probability)	Online Tool
Predicted LD_50_ (mg/Kg) in rodents	159	ProTox-II
Predicted toxicity class (according to GHS)	3	
Hepatotoxicity	inactive (0.70)	
Carcinogenicity	active (0.71)	
Immunogenicity	inactive (0.51)	
Mutagenicity	inactive (0.53)	
Cytotoxicity	inactive (0.98)	
Aryl hydrocarbon receptor	active (0.84)	Nuclear receptor signaling and stress response pathways (ProTox-II)
Androgen receptor	inactive (0.99)	
Androgen receptor ligand-binding domain	inactive (0.72)	
Aromatase	inactive (0.88)	
Estrogen receptor α	active (0.69)	
Estrogen receptor ligand-binding domain	active (0.86)	
Peroxisome-proliferator activated receptor γ	inactive (0.98)	
Nrf2/ARE	inactive (0.98)	
Heat Shock Factor Response Element	inactive (0.98)	
Mitochondria Membrane Potential	active (0.82)	
p53	inactive (0.97)	
ATPase family AAA domain-containing protein 5	inactive (0.77)	
Carcinogenicity	no	CarcinoPred-EL
Mutagenic	yes	OSIRIS Property Explorer
Tumorigenic	yes	
Irritant	yes	
Reproductive effective	yes	
Ames test	mutagen non-mutagen (pkCSM)	PreADMET
Carcino_mouse	no	
Carcino_rat	yes	
Algae_at	0.0495876	
Daphnia_at	0.200841	
hERG inhibition	medium risk	
Medaka_at	0.0667019	
Minnow_at	0.0294925	
TA100_10RLI	no	
TA100_NA	yes	
TA1535_NA	no	
Max. tolerated dose (human) (log mg/Kg/day)	0.973	pkCSM
hERG I inhibitor	no	
hERG II inhibitor	yes	
Oral rat acute toxicity (LD_50_ mol/Kg)	2.111	
Oral rat chronic toxicity (NOAEL log mg/Kg_bw/day)	3.014	
Hepatotoxicity	no	
Skin sensitization	no	
*T. pyriformis* toxicity (log µg/L)	0.341	
Minnow toxicity (log mM)	0.976	

GHS: globally harmonized system of classification and labeling of chemicals, rev. 8; NOAEL: no observed adverse effect level.
